# Elevated temperature and pressure performance of water based drilling mud with green synthesized zinc oxide nanoparticles and biodegradable polymer

**DOI:** 10.1038/s41598-025-96900-z

**Published:** 2025-04-08

**Authors:** Milad Khashay, Mohammad Zirak, James J. Sheng, Tarek Ganat, Ehsan Esmaeilnezhad

**Affiliations:** 1 Department of Petroleum Engineering, Hakim Sabzevari University, Sabzevar, Iran; 2 Department of Physics, Hakim Sabzevari University, Sabzevar, Iran; 3 Bob L. Herd Department of Petroleum Engineering, Texas Tech University, Lubbock, TX 79409 USA; 4 Petroleum and Chemical Engineering Department, Sultan Qaboos University, 123 Al Khodh, Sultanate of Oman

**Keywords:** Environmentally-friendly WBMs, Green-synthesized ZnO NPs, Natural biodegradable TG, HTHP filtration, Solid Earth sciences, Energy science and technology, Engineering, Nanoscience and technology

## Abstract

Water-based mud (WBM) faces challenges in high-temperature, high-pressure (HTHP) conditions due to fluid loss and property degradation. Enhancing eco-friendly drilling fluids with optimal rheology is crucial for sustainable, cost-effective, and environmentally safe drilling operations. This study formulated a WBM using green-synthesized zinc oxide (ZnO) nanoparticles (NPs, ~ 45 nm) and tragacanth gum (TG), a biodegradable natural polymer. The synthesized ZnO NPs were comprehensively characterized using energy-dispersive X-ray spectroscopy (EDS), field-emission scanning electron microscopy (FE-SEM), Raman spectroscopy, X-ray photoelectron spectroscopy (XPS), and thermogravimetric analysis (TGA/DTG) to determine their structural, morphological, and chemical properties. Rheological properties, including flow behavior index (n), consistency index (K), plastic viscosity (PV), and yield point (YP), were analyzed at 25, 50, and 75 °C using the Bingham-plastic and Power-law models. The accuracy of the model was validated using Analysis of Variance (ANOVA), which assessed the significance of the results. Additionally, Design Expert software was utilized to optimize the concentrations of TG and ZnO for elevated temperature applications. Moreover, the response surface methodology (RSM) results were evaluated by reporting the R^2^ and accuracy metrics, confirming the strong correlation between predicted and actual values, which demonstrates the model’s robustness. Three optimal samples underwent HTHP filtration tests at 120 °C and 500 psi. The ideal formulation of 750 ppm TG and 0.25 wt% ZnO NPs improved PV by 27.84%, YP by 43.16%, reduced fluid loss by 54.16%, and mud cake thickness by 25%. The optimized sample showed superior performance, with a ‘K’ of 56.12 cp and a ‘n’ of 0.2272, ensuring effectiveness under HTHP conditions. This sustainable formulation reduced environmental contamination risks and drilling fluid consumption while enhancing operational efficiency.

## Introduction

The energy industry is driven by increasing energy demand and the need to improve technologies for exploration and drilling in more challenging formations due to the gradual exhaustion of conventional petroleum reserves. Extracting oil and gas from deeper formations under high-temperature, high-pressure (HTHP) conditions has become more complicated, leading to higher costs and more significant environmental risks. Many of these challenges are linked to the circulation system used in drilling operations, where the drilling fluid is vital^1,2^. The performance of drilling mud strongly depends on its rheological and filtration properties. The complex physical, chemical, and electrochemical changes in drilling mud composition at elevated temperatures and pressure make predicting these characteristics more challenging^3^. Conventional drilling mud formulations frequently include chemical additives that exhibit varying degrees of toxicity. Consequently, a poorly designed drilling mud formulation under HTHP conditions can cause these toxic components to invade the surrounding formations, leading to formation damage and potentially reducing the efficiency of the drilling operation. Furthermore, this invasion can increase environmental risks by contaminating nearby ecosystems and water sources. Developing a comprehensive drilling strategy and selecting the most suitable drilling fluid with optimal properties are crucial for successful drilling operations in HTHP environments. Additionally, choosing environmentally friendly drilling fluids can help mitigate the associated environmental risks and contribute to more sustainable practices. Drilling muds are categorized into water-based^4^, oil-based^5^, and synthetic-based types^6^, with water-based muds (WBMs) being more cost-effective^7^ and environmentally friendly^8,9^. They also offer better cooling^10^, cutting removal^11^, and faster penetration rates than the other types^12^. Formulating an efficient WBM requires careful consideration of its additives to improve rheological and filtration properties under HTHP conditions while minimizing environmental risks^13,14^. To address these challenges, experimental research has focused on using less toxic and natural additives to enhance the rheological characteristics and reduce filtration loss^9,15,16^.

Over the past few decades, nanotechnology has revolutionized nearly every industry. Nanoparticles (NPs) are now being utilized and explored for applications in the petroleum industry, particularly in developing WBMs^17^. Additionally, nanofluids have gained significant attention in enhanced oil recovery (EOR) due to their ability to improve sweep efficiency, alter wettability, reduce interfacial tension, and modify fluid viscosity, ultimately enhancing oil displacement and recovery^18,19^. Another advantage of using NPs is their ability to significantly enhance the thermal conductivity of drilling fluids, surpassing that of polymeric additives^20,21^. Adding NPs to drilling fluids significantly enhances their rheological stability across temperatures ranging from 25 to 80 °C^22^. Numerous studies have explored using NPs as additives in drilling fluids, including Bismuth Ferrite^23^, Hydrophilic Gilsonite^24^, Cupric Oxide^25^, Zinc Oxide (ZnO)^26^, Zinc Titanate^27^, and Magnesium Aluminum Silicate^28^. Hydroxyapatite NPs improved cuttings transport in WBM, increasing viscosity by 168% and reducing fluid loss. At 2.0 g concentration, hydroxyapatite NPs enhanced the cuttings transport ratio by 28–38.6%^17^. Biogenic copper oxide NPs, synthesized using *Colocasia esculenta* extract, improved drilling fluid performance by enhancing lubricity (27%) and filtration (48%). They formed a thin lubricating film, reducing friction and maintaining rheological stability under high temperatures^29^. Silica and copper oxide NPs have been used to enhance non-damaging drilling fluids and bentonite-based drilling fluids properties. Silica NPs reduced filtrate loss by 31%, acting as a thickener in non-damaging drilling fluids and a thinner in bentonite-based drilling fluids^25^. A biosynthesized ZnO nanofluid in WBM enhanced rheological properties (PV: 67.9 cp, YP: 29.71 Pa) and improved wettability by reducing the contact angle by over 51%. It minimized formation damage by creating a thin, impermeable mud cake^30^. ZnO is an ideal NPs for drilling fluids, attributed to its high heat capacity^31^, excellent thermal conductivity^32^, minimal thermal expansion^33^, and elevated melting point^34^. Moreover, the outstanding colloidal properties of ZnO are likely to provide stability to the colloidal mechanisms of other NPs. Given that ZnO can generate thick drilling fluids with elevated YPs under HTHP conditions, it is advisable to use ZnO in low quantities within drilling fluids^26,35^. Adding ZnO NPs improved the YP of WBMs, resulting in a 61.54% increase at 40 °C and a 60% increase at 80 °C. This demonstrates their potential to enhance fluid performance across varying temperatures^36^. The enhancements in rheological and filtration properties observed using ZnO NPs under HTHP conditions were significant. Drilling fluid formulations containing specific ratios of water and montmorillonite, combined with a ZnO–clay nanocomposite, exhibited excellent stability and maintained consistent rheological properties in HTHP environments^35^. These findings indicate that selecting ZnO NPs is a beneficial choice as an additive for drilling fluids. Recent advances in nanotechnology have led to the development of various methods for synthesizing these NPs. These approaches include biological, chemical, and physical methods, enabling the production of NPs with different sizes, surface properties, functions, and behaviors. NPs can be made from various materials, such as metals, semiconductors, ceramics, metal oxides, and polymers^37,38^. Biological methods, known as green technology, offer an eco-friendly, low-toxicity, and economical approach for synthesizing NPs. These techniques align with green technology principles, making them a more sustainable option for NPs synthesis^39^. Biological systems such as bacteria, fungi, and plant extracts are utilized in these methods to synthesize metal and metal oxide NPs. Using plant extracts is a highly effective, clean, and environmentally friendly approach^40^.

Biopolymers have gained significant attention in the petroleum industry due to their environmentally friendly nature^41–43^ and multifunctional applications^18,44^, including enhanced oil recovery (EOR)^19,45,46^, drilling operations, and wellbore stability. In EOR, biopolymers improve sweep efficiency and reduce interfacial tension, facilitating higher oil recovery^47–49^. In drilling fluids, they enhance rheological properties, provide effective fluid loss control, and improve cuttings suspension, making them valuable additives for both conventional and unconventional reservoirs. Recently, drilling fluids have been augmented with different natural biopolymer gums to improve their filtration and rheological properties^50–52^. Polymeric additives provide an advanced solution due to their thixotropic and shear-thinning properties^18,53,54^. They also provide adjustable yield stress, which can be modified to suit various flow and shear conditions^51,55^. Significant changes in mud weight, YP, PV, apparent viscosity, and gel strength were observed due to changes in the concentration of the locally selected biopolymers^56^. The HTHP filtrate loss of WBMs was reduced by 69.2% by adding five percent of a biodegradable cellulosic material called Grewia Optiva to the reference mud at 100 °C and 500 psi^50^. Biopolymers such as dually modified starch are added to drilling fluids to improve borehole stability and reservoir productivity^55,57^. Due to its high thermal resistance, acidity, and extended shelf life, tragacanth gum (TG)^58^, a bio-polymer, is used extensively as an emulsifier, thickening agent, and stabilizer in different sectors^58^. The laboratory study reveals that TG is an effective viscosity modifier, while the mud filtrate minimally impacts formation damage^59^. It was found through laboratory investigation that the TG and bentonite combination results in favorable rheological properties with less formation damage^60^. The apparent viscosity, PV, and YP values were increased by TG across all concentrations^61^.

However, the HTHP conditions in deep wells demand more excellent thermal stability from natural polymers used in drilling fluids. These polymers tend to degrade when exposed to high temperatures for extended periods during drilling operations. A key focus of research is improving the thermal resistance of polymers and integrating innovative materials into drilling fluids^53,62,63^. Recent studies have investigated combining NPs with polymeric additives in drilling fluids to improve their rheological and filtration characteristics under HTHP conditions^62,64^. In the presence of titanium dioxide NPs, polymeric WBMs showed increased thermal stability, improved shale recovery to 97.2%, and reduced filtrate loss by 27%^65^. Incorporating a high-temperature synthetic polymer with carbon nanotubes into WBM enhanced rheology by 14% and decreased filtrate volume by 25%^66^. Adding 0.25 wt% of polymers and 0.25 wt% of NPs to the WBM significantly improved its filtration and rheological characteristics, including YP and shear viscosity, at temperatures between 25 and 85 °C^67^. The polymer-based silica NPs composite, employed as a micro-nano additive in drilling fluids, demonstrated outstanding characteristics, including enhanced thermal stability, improved rheological performance, reduced fluid loss, and superior lubricity^68^. Titanium dioxide and Silica dioxide NPs substantially enhance the filtration and rheological characteristics of polymeric drilling fluids across a temperature range of 25 to 75 °C, with Silica dioxide NPs showing a more pronounced effect^53^. In examining the influence of various factors on rheological properties, the optimal conditions were identified as 0.82 wt% for the natural polymer starch, 0.2 wt% for ZnO NPs, and 65 min of ultrasonic time. The analysis indicated that the ZnO NPs had the most substantial effect on the rheological properties^20^. The individual and combined effects of ZnO NPs and hydrophobically modified associative polymer on drilling fluid properties were studied, focusing on rheology, filter cake, and shale inhibition. Increasing the ZnO NPs and the polymer concentrations improved the rheological characteristics of the WBMs^69^. Incorporating a ZnO NPs-acrylamide composite enhanced lubricity by 25% at 65 °C and decreased the filtrate loss by 14%. Under elevated temperature and pressure conditions (500 psi, 120 °C), there was a modest reduction in filtrate loss volume, and shale swelling decreased from 16 to 9%^70^. Incorporating ZnO NPs and TG polymer in drilling mud formulations was studied, leading to notable enhancements in rheological and filtration properties under atmospheric pressure and room temperature conditions. This foundational study established the potential of these materials to enhance drilling fluid performance, particularly in terms of viscosity control and filtration loss reduction^26^.

The synergistic effect of ZnO NPs and TG polymers under HTHP conditions has not been extensively investigated. Previous studies, including our own, demonstrated the efficacy of this combination under ambient temperature and atmospheric pressure. However, a clear gap exists in understanding their performance at elevated temperatures (50 °C and 75 °C) and high-pressure conditions. Additionally, unlike our previous work, where ZnO NPs were synthesized via a chemical route, this study employs an environmentally friendly green synthesis method, contributing to the development of more sustainable drilling fluid additives. This research aims to bridge these gaps and provide insights into the thermal stability and filtration performance of these additives under realistic wellbore conditions.

A series of drilling fluid formulations were prepared using Design Expert software and the central composite design (CCD) method to explore the effects of these eco-friendly additives on elevated temperature rheology and HTHP filtration characteristics. This study investigates the thermal stability of drilling mud, focusing on the novel use of green-synthesized ZnO NPs and TG polymer to enhance mud performance under HTHP conditions, providing a sustainable and effective solution for challenging drilling environments.

## Materials and methods

This study utilized zinc acetate dihydrate (ZnAc⋅2H_2_O), local bentonite, sodium carbonate, deionized (DI) water, and TG, sourced from Merck Co. and local suppliers^26^. This work employed two main approaches, incorporating both the green synthesis of ZnO NPs using TG polymer and Design Expert software for experimental design. TG, a natural and biodegradable polymer, was utilized due to its well-established eco-friendly properties as documented in prior research^71^. Additionally, the ZnO NPs were synthesized using TG as a stabilizing agent through a green synthesis method, which avoided the use of hazardous chemicals and aligned with sustainable practices^72,73^. This approach not only enhances the functionality of the drilling fluid but also ensures its environmental compatibility, reinforcing the claim of eco-friendliness for the developed formulation. The integration of TG and green-synthesized ZnO NPs highlights a step toward more sustainable and environmentally responsible drilling fluid technologies. Additionally, Design Expert software optimizes the experimental design process, minimizing resource consumption by reducing the number of experiments required. This results in more efficient use of materials and energy, supporting sustainable research practices.

### Green synthesis of ZnO NPs

ZnO NPs were green-synthesized using the sol–gel method. ZnAc⋅2H_2_O was utilized as a precursor in ZnO NPs synthesis. Initially, 0.15 g of TG was dissolved in 40 ml of DI water and stirred for 80 min at 70 °C. Afterward, 1.5 g of zinc salt was introduced into the resulting gel. The mixture was transferred to a water bath held at a steady temperature of 75 °C and was stirred continuously for 12 h. This process produced a white resin, followed by calcination in air at 500 °C for three hours, yielding ZnO NPs^73–75^.

### Experimental design and sample preparation

Employing response surface methodology (RSM), made assessing multiple factors on response variables more efficient, and reduced time and cost. RSM helps minimize environmental impact by optimizing processes with fewer experimental runs, reducing resource consumption and waste generation. It enables efficient use of materials and energy, contributing to sustainable and environmentally friendly practices. Based on previous studies, sensitivity analysis determined that the ZnO NPs concentrations (A), TG polymer concentrations (B), and elevated temperatures (C) are significant factors influencing the responses. The evaluated parameters comprised PV and consistency index (K) measured in centipoise, YP in Pa, and the dimensionless flow behavior index (n). Based on prior research, ZnO NPs concentrations of (0, 0.25, 0.5, 0.75, and 1 wt%) and TG polymer concentrations of (0, 250, 500, 750, and 1000 ppm) were chosen for investigation at three different temperatures (25, 50, and 75 °C)^3,26^. The temperature range of 25–75 °C was selected to evaluate the viscosity and rheological behavior of WBMs containing ZnO NPs and TG polymers. This range was chosen based on benchmarks from related studies and serves as a preliminary step to identify optimal formulations for subsequent HTHP filtration tests^22^. This approach conserves resources, as HTHP tests are both time-intensive and costly. To ensure transparency in the optimization process, a summary of the selected maximum and minimum values of the input variables is presented in Table 1. These values were carefully chosen based on experimental constraints and the CCD framework to achieve optimal response conditions.

**Table 1:** Selected maximum and minimum values of input variables used in the CCD for optimization.

Factor	Name	Units	Type	Minimum	Maximum	Coded low	Coded high
A	ZnO NPs	wt%	Numeric	0	1	− 1 ↔ 0.25	+ 1 ↔ 0.75
B	TG	ppm		0	1000	− 1 ↔ 250.00	+ 1 ↔ 750.00
C	Temperature	℃		25	75	− 1 ↔ 25.00	+ 1 ↔ 75.00

Table 2 outlines the tests conducted, with each experiment repeated in triplicate to ensure accuracy. The data were analyzed using RSM and CCD, which facilitated the development of a mathematical model to illustrate the effects and interactions of the variables. The impact of different parameters was evaluated using analysis of variance (ANOVA) to identify the optimal mud fluid formulation. This formulation was then validated through statistical analysis and experimental testing. Following API standard 13B-2^76^ for drilling mud preparation and testing, a sodium carbonate solution mixed with bentonite was prepared, and stock solutions of ZnO NPs and TG were also made. The final WBMs were formulated by mixing all the components and then homogenized using a vortex shaker set at 2500 rpm to ensure uniform dispersion and reduce agglomeration (Table 3). A comprehensive description of the procedure can be found in our earlier research^26^.

**Table 2:** Range of independent variables experimental runs by CCD.

Std. No	Run	Factors		
		(A) ZnO Conc. (wt%)	(B) TG Conc. (ppm)	(C) Temperature (°C)
14	1	0.5	500	75
11	2	0.5	0	50
19	3	0.5	500	50
4	4	0.75	750	25
5	5	0.25	250	75
9	6	0	500	50
2	7	0.75	250	25
12	8	0.5	1000	50
1	9	0.25	250	25
17	10	0.5	500	50
13	11	0.5	500	25
8	12	0.75	750	75
10	13	1	500	50
16	14	0.5	500	50
15	15	0.5	500	50
7	16	0.25	750	75
6	17	0.75	250	75
18	18	0.5	500	50
3	19	0.25	750	25

**Table 3:** Mud sample formulation.

Final mud volume (cc)	Sodium carbonate (g)	Bentonite (g)	ZnO NPs (wt%)	TG polymer (ppm)
350	1.5	22.5	0, 0.25, 0.5, 0.75, 1	0, 250, 500, 750, 1000

## Characterizations and measurements

In this section, the green-synthesized ZnO NPs and drilling fluid samples are subjected to various characterizations and measurements to assess their structural, rheological, and filtration properties. These evaluations are essential for understanding the influence of TG and ZnO NPs on the overall performance of the WBMs, especially under HTHP conditions.

### Structural characterizations

The morphology of the NPs and their energy-dispersive X-ray spectroscopy (EDS) mapping were examined using field-emission scanning electron microscopy (FE-SEM). Raman spectroscopy was performed using a high-resolution UniDRON-A confocal microscope Raman/PL spectroscopy system from Korea, equipped with a 532-nm edge laser. ZnO NPs were also analyzed using X-ray photoelectron spectroscopy (XPS) in an ultra-high vacuum chamber with a monochromatic Al Ka X-ray source at 1486.6 eV. The binding energies (BE) were calibrated for charge shifts by referencing the C 1s peak of graphitic carbon at 285 eV. Thermogravimetric analysis and its derivative (TGA/DTG) for the ZnO NPs were carried out using a TGA STA6000. The analysis involved heating from room temperature up to 600 °C at 15 °C per minute.

### Rheological measurements

The rheological properties of the mud samples at elevated temperatures were evaluated employing a Brookfield RST-CC Rheometer, which provided precise measurements of the fluid’s behavior under various temperature conditions. The Bingham Plastic and Power-Law models were selected due to their widespread use in drilling fluid characterization and their proven effectiveness in evaluating key parameters such as viscosity and shear-thinning behavior. The decision not to use the Herschel–Bulkley model was based on its inclusion of an additional yield stress parameter, which was not central to the scope of this study. It is acknowledged that a comprehensive mathematical model-fitting approach, including the Herschel–Bulkley model, may be incorporated in future research for further refinement of the rheological characterization.

After data collection, modeling was performed using the Power-law and Bingham-plastic rheological models.

Bingham-plastic^77^:

1 $$\uptau ={\uptau }_{0}+{\upmu }_{p}\dot{\upgamma }$$

Power-law^78^:

2 $$\uptau =K{\dot{\upgamma }}^{n}$$

where, $\uptau$ represents shear stress, ${\uptau }_{0}$ is the YP, ${\upmu}_{p}$ denotes PV, and $\dot{\upgamma }$ is the shear rate. ‘K’ indicates the consistency index, whereas ‘n’ signifies the flow behavior index.

Nineteen distinct drilling fluid samples were prepared by mixing varying concentrations of NPs with polymer, as detailed in Table 2. These samples included five central points, and the combinations were determined using Design Expert software. The rheological properties were assessed at 25, 50, and 75 °C under atmospheric pressure (1 atm), with shear rates up to 1500 s^−1^.

### HTHP filtration measurements

Bentonite starts to deteriorate at temperatures around 120 °C, leading to increased filtrate loss and reduced effectiveness in hole cleaning^3^. Conducting the test at the in-situ down-hole temperature and pressure provided precise measurements of filtration characteristics^65,70^. The filtration experiment followed the API RP 13B-1 standard procedure^76,79^, and HTHP filtration characteristics were examined using an OFITE HTHP filter press, conducted at a temperature of 120 °C and a differential pressure of 500 psi to determine fluid loss and mud cake thickness. Filtrate volume was recorded over 30 min, and the thickness of the mud cake was measured using a caliper^66^.

## Results and discussion

The green-synthesized ZnO NPs’ structural characterization and prepared eco-friendly WBMs’ rheological and filtration characteristics, namely filtrate loss and filter cake thickness, have been assessed and are discussed in the following sections.

### ZnO NPs characterization

**Fig. 1:**
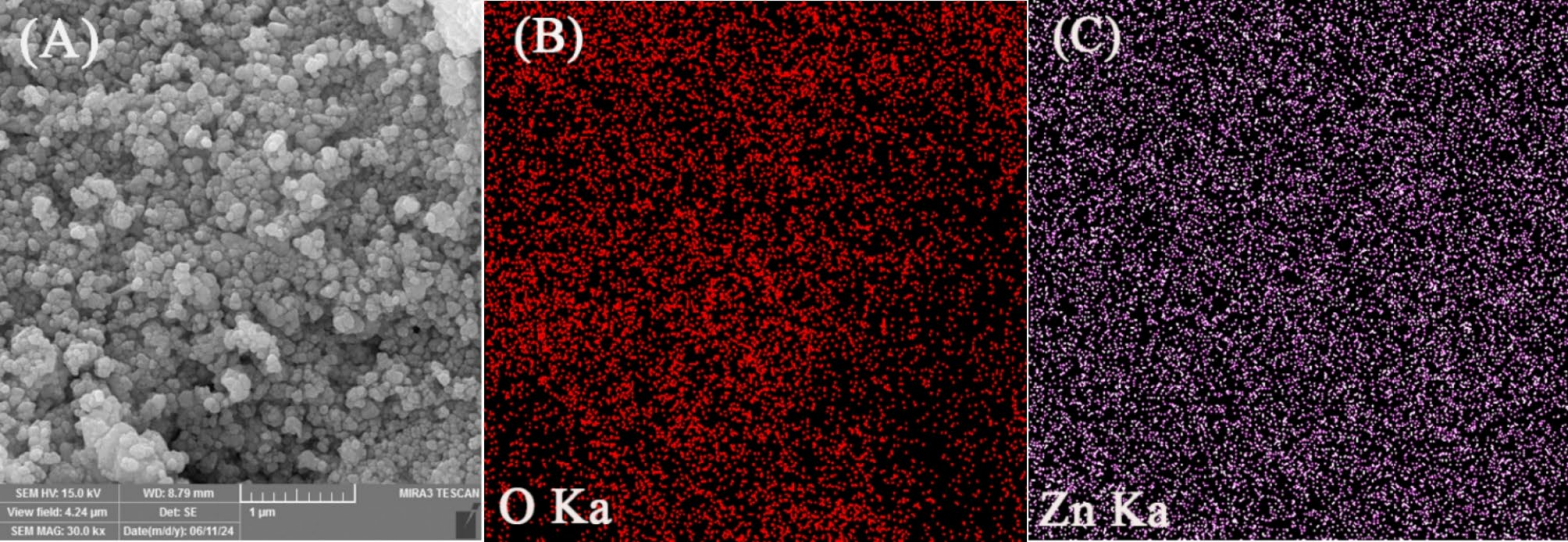
FESEM images of synthesized ZnO NPs (**A**) and elemental EDS mapping of oxygen (**B**) and zinc (**C**) elements obtained from ZnO NPs surfaces.

The micro-morphology of the green-synthesized ZnO nanostructures was assessed using FE-SEM (Fig. 1A). The analysis revealed that the nanostructures have spherical shapes with sizes ranging from 20 to 60 nm. Such highly symmetric morphology is beneficial for improving rheological characteristics. EDS was employed to assess the elemental composition of the ZnO NPs. Figure 1B and C are elemental EDS mappings representing the surface distribution of O and Zn elements, respectively. The maps indicate that Zn and O elements are uniformly distributed throughout the ZnO powder (Fig. 1B and C). EDS spectra and FESEM images reveal the incorporation of Zn and O elements in the spherical NPs, confirming that the sample produced by the sol–gel method consists of pure ZnO phases.

**Fig. 2:**
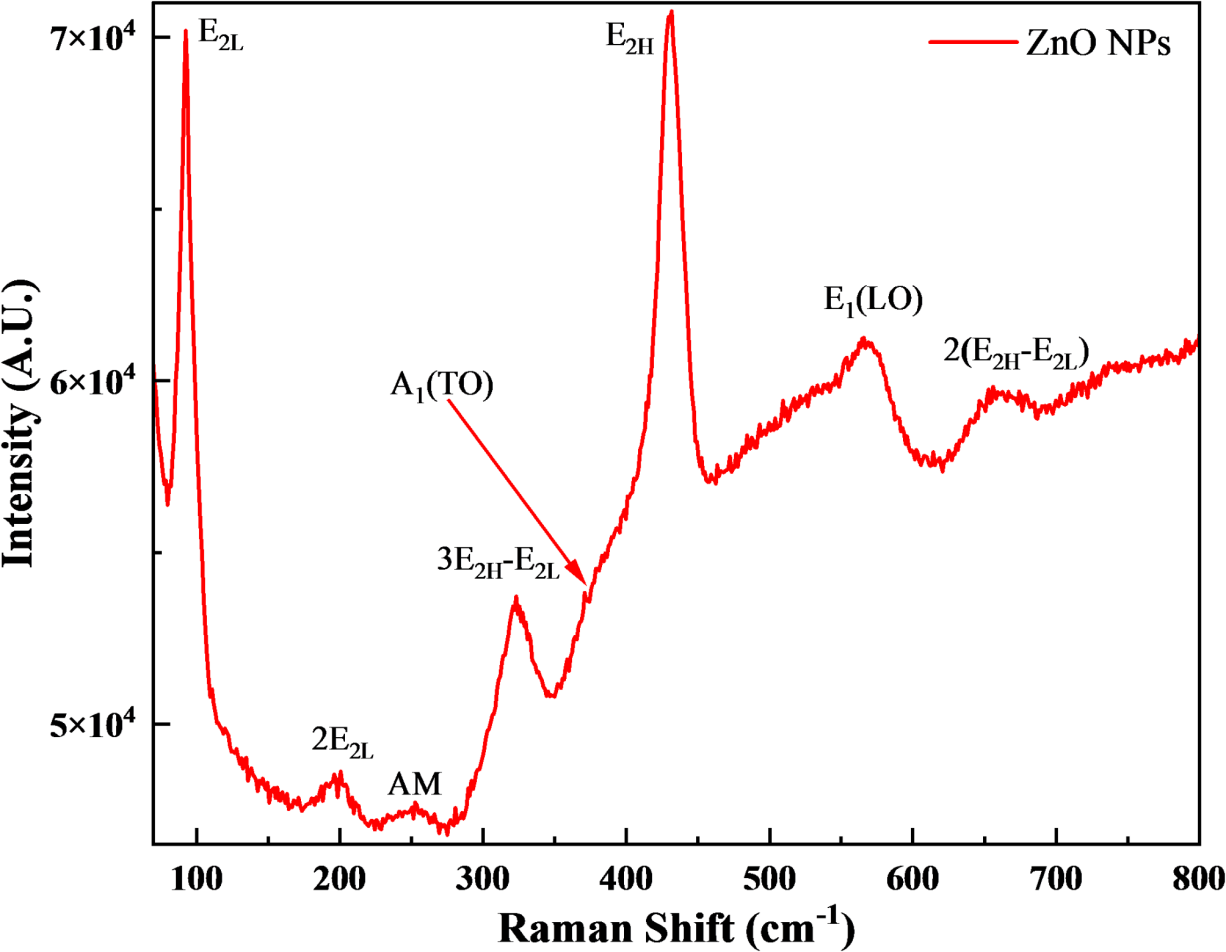
Raman spectrum of synthesized ZnO NPs.

Raman spectroscopy was used to analyze the crystallinity of the ZnO NPs, which detects variations in the crystal lattice and associated defects. The ZnO crystal structure is wurtzite (hexagonal), classified under the ${C}_{6v}^{4}$ space group. The crystal has a primitive cell containing two formula units and features atoms at sites with ${C}_{3v}$ symmetry. Group theoretical analysis indicates eight distinct sets of optical phonon modes at the zone center. In this arrangement, the A_1_ and E_1_ modes are polar and separate into transverse optical phonons (A_1T_ and E_1T_), while the E_2_ mode includes two Raman-active phonons, the low-frequency E_2L_ and the high-frequency E_2H_^80^. Figure 2 displays the Raman-scattering spectrum of the ZnO NPs, measured at 40 °C using a 532 nm laser as the excitation source. A prominent, sharp peak labeled E_2_ at 432 cm⁻^1^ was observed, indicating a Raman-active optical phonon mode characteristic of the wurtzite hexagonal phase of ZnO. Additional peaks commonly observed are around 93 cm⁻^1^, referred to as E_2_(Low), and the peak at 323 cm⁻^1^, corresponds to a second-order Raman mode due to zone-boundary phonons (3E_2H_–E_2L_). Another notable peak at 370 cm⁻^1^ is A_1_(TO). The higher intensity and sharpness of the E_2_ mode peak at 432 cm⁻^1^, in comparison to the other peaks observed, indicate that the ZnO NPs exhibit a wurtzite hexagonal phase with high crystallinity^81^.

**Fig. 3:**
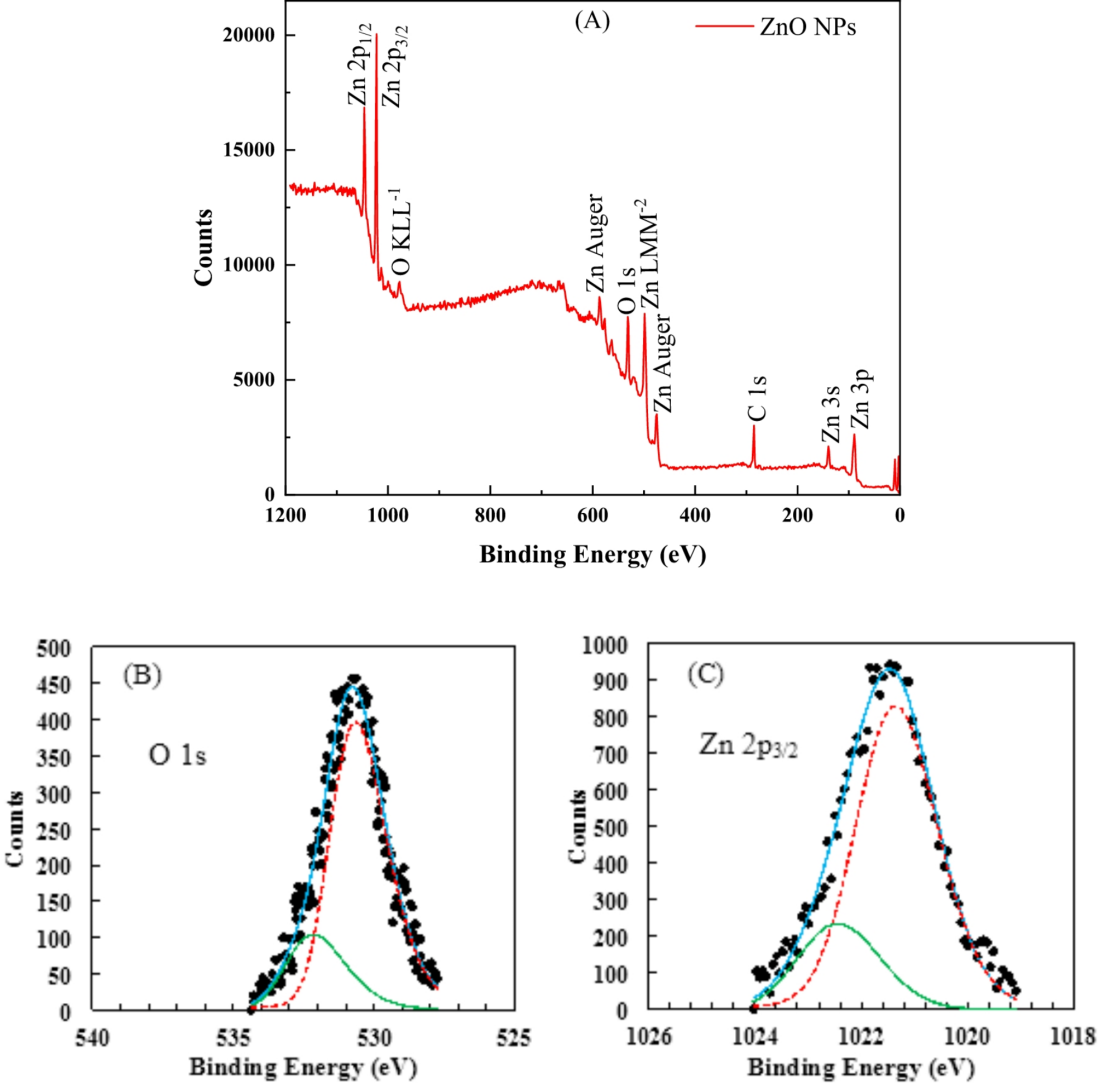
XPS survey spectrum (**A**) and corresponding high resolution XPS spectra of O 1s (**B**) and Zn 2p_3/2_ (**C**) core levels obtained for synthesized ZnO NPs.

The surface chemical composition of green-synthesized ZnO NPs was examined via XPS. Figure 3A shows the survey XPS spectrum for ZnO NPs. Clear signals related to O and Zn elements can be observed. The C 1s peak arises from environmentally adsorbed carbon species such as CO_2_, and CO. The survey spectrum revealed that the ZnO NPs contain only zinc and oxygen elements with no trace of impurities. ZnO NPs produced by this simple synthesis method appear to have high purity. The chemical composition was investigated more deeply via high-resolution XPS spectra. The results for the O 1s and Zn 2p_3/2_ core levels are shown in Fig. 3B and C, respectively. O 1s signal was fitted with two peaks at BE of 530.63 and 532.15 eV, associated with Zn–O and Zn–O–H bonds, respectively^82,83^. The –OH bonds on the ZnO NPs can be verified by the Zn 2p_3/2_ signal fitted with two peaks (Fig. 3C). The primary peak is at BE of 1021.34 eV, which is related to electrons of the Zn–O bond. Another peak with BE of 1022.44 eV is assigned to the Zn–OH bond, in agreement with the O 1s XPS signal. Others have also reported detecting hydroxide species on the surfaces of ZnO nanostructures prepared using solution-based methods^83,84^. The hydroxide species (Hydroxyl (–OH) groups) on the surface of ZnO NPs influence their adhesion and wettability characteristics^85–87^, These functional groups are highly polar, allowing them to form strong hydrogen bonds with water molecules. Therefore, the presence of –OH groups on the surface of ZnO NPs makes them more hydrophilic, meaning they have a greater affinity for water. In addition, hydroxide species increases the surface energy of the NPs promoting the spreading of water on the surface, and hence to better wetting is obtained. Moreover, these hydroxyl groups contribute to increased particle adhesion through increasing the hydrogen bonding and van der Waals forces. Surface wettability and adhesion of the NPs are essential factors for drilling fluid stability and performance. These properties enhance particle dispersion, improve suspension stability, and modify interactions with bentonite, leading to better rheological control and reduced filtration loss.

**Fig. 4:**
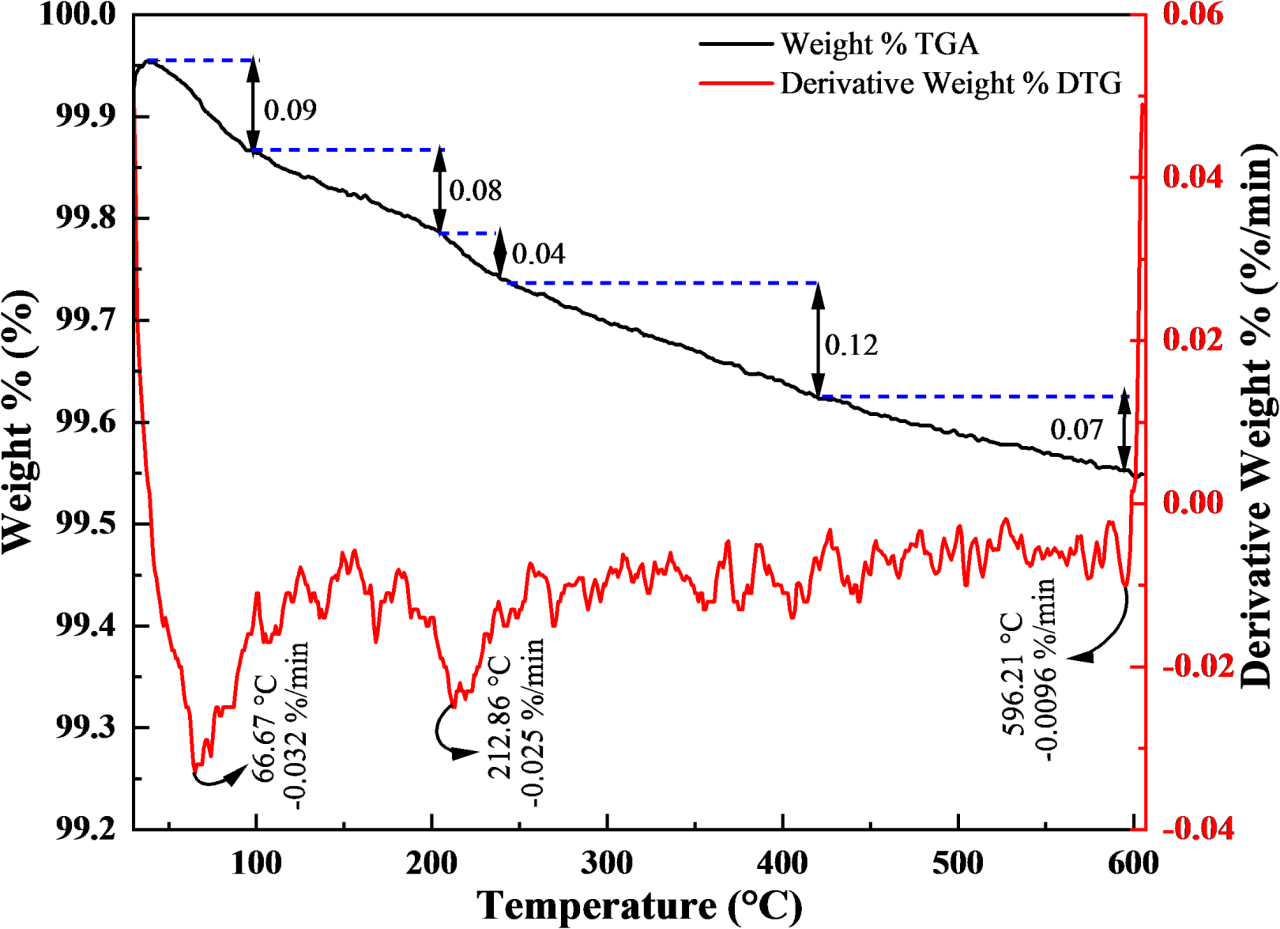
TGA/DTG curves of the ZnO NPs synthesized by the green sol–gel method.

The thermal resistance of the synthesized ZnO NPs was examined using TGA-DTG analysis. Figure 4 presents the TGA-DTG results of the ZnO NPs. The initial decomposition losses, ranging from 37.62 to 204.45 °C, are ascribed to the loss of water adsorbed on the NPs. The second phase of decomposition, spanning from 204.45 to 420.18 °C, is attributed to the volatilization and combustion of organic species in the sample^88^. The overall weight reduction observed for ZnO NPs is about 0.45%, demonstrating their high thermal resistance. This minimal weight loss underscores the high purity of the ZnO NPs, which aligns with the findings presented in earlier research^26,88,89^. The high thermal resistance of the synthesized ZnO NPs is attributed to their strong Zn–O bonds and stable crystalline structures. The presence of these bonds was confirmed by Raman spectroscopy, and XPS analyses. ZnO NPs are synthesized at temperatures up to 500 °C, leading to the formation of thermally stable crystal phases which was confirmed by TGA-DTG analysis. These structural properties prevent degradation at elevated temperatures, ensuring that ZnO NPs contribute to the stability of rheological and filtration properties in drilling fluids under HTHP conditions.

### Rheological characteristics

The variables and their interactions are represented with coded factors, where (A) corresponds to ZnO NPs concentration, (B) to TG concentration, and (C) to temperature variation. Equations (3) through (6) represent the final formulations in terms of coded factors. These empirical models, detailed in Table 4, show how the input variables affect the desired responses. The efficacy of these models in predicting fluid rheological behavior has been demonstrated. The coefficients in the regression equations derived from ANOVA provide insights into how the predictor variables (A, B, C) and their interactions influence the response variables (YP, PV, ‘n’, and ‘K’). Positive coefficients indicate a direct enhancement of the respective response, while negative coefficients signify an inhibitory effect. The interaction terms (AB, AC, BC, ABC) highlight synergistic or antagonistic effects between variables, influencing the fluid’s rheological and filtration behavior. The trends observed align with the physicochemical mechanisms governing drilling fluid performance, emphasizing the role of TG and ZnO NPs in optimizing viscosity, yield stress, and shear-thinning properties under HTHP conditions. Consequently, the equations enable accurate assessment of elevated temperature, ZnO NPs concentration, and TG polymer level to obtain the desired responses. Furthermore, these equations simplify the process of optimization when required.

**Table 4:** Final equations in terms of coded factors for responses.

Responses	Predicted equations	Eq.
YP	$$\begin{aligned} & 3.85 - 0.8169 \times A + 0.0681 \times B + 0.3380 \times {\text{C}} - 0.6337 \\ & \quad \times {\text{AB}} + 0.0438 \times {\text{AC}} + 0.0563 \times {\text{BC}} - 0.7188 \times {\text{ABC}} \\ \end{aligned}$$	(3)
PV	$$\begin{aligned} & 6.76 - 0.6938 \times A + 0.6437 \times B - 2.15 \times {\text{C}} - 0.7625 \\ & \quad \times {\text{AB}} - 0.2875 \times {\text{AC}} + 0.0875 \times {\text{BC}} - 0.0125 \times {\text{ABC}} \\ \end{aligned}$$	(4)
‘n’	$$\begin{aligned} & 0.1962 - 0.0017 \times A - 0.0100 \times B + 0.0177 \times {\text{C}} \\ & \quad - 0.0066 \times {\text{AB}} - 0.0133 \times {\text{AC}} - 0.0008 \times {\text{BC}} - 0.0016 \times {\text{ABC}} \\ \end{aligned}$$	(5)
‘K’	$$\begin{aligned} & 35.81 - 7.72 \times A - 2.23 \times B + 12.72 \times {\text{C}} - 1.97 \\ & \quad \times {\text{AB}} + 0.6513 \times {\text{AC}} - 1.10 \times {\text{BC}} - 2.10 \times {\text{ABC}} \\ \end{aligned}$$	(6)

The acquired data were analyzed using the rheological models to clarify the fundamental interconnections among the variables and their responses. Table 5 provides the fitting parameters for the equations, and the adjusted R-squared (Adj R^2^) values demonstrate that the rheological models accurately represent the experimental data. Generally, the Power Law model demonstrated a stronger correlation with WBMs than the Bingham model^90^. Table 5 reveals that the Adj R^2^ values for some samples are close to one, demonstrating a solid alignment between the WBMs and the rheological models. The flow behavior of the WBMs appears to have been significantly impacted by the additives, resulting in a more substantial alignment between the experimental data and the predictions made by the rheological models.

**Table 5:** Parameters values of samples using Power low, and Bingham fluid models.

Std. No.	Run	Temperature (°C)	Responses					
			Power low			Bingham		
			‘K’	‘n’	Adj R^2^	YP (Pa)	PV (cp)	Adj R^2^
14	1	75	50.45	0.2148	0.94	4.21	3.5	0.78
11	2	50	36.84	0.2149	0.91	3.66	5.7	0.73
19	3	50	34.85	0.1951	0.93	3.82	6.5	0.76
4	4	25	13.16	0.1727	0.84	2.78	8.0	0.78
5	5	75	56.75	0.2323	0.97	3.51	4.5	0.89
9	6	50	49.68	0.1983	0.99	5.48	7.5	0.99
2	7	25	17.29	0.2011	0.86	2.61	8.0	0.72
12	8	50	32.12	0.1751	0.94	3.98	7.5	0.76
1	9	25	35.83	0.1676	0.95	4.50	8.0	0.97
17	10	50	36.25	0.1954	0.96	3.79	6.7	0.78
13	11	25	20.15	0.1961	0.85	3.42	9.5	0.86
8	12	75	32.30	0.1808	0.89	2.19	3.7	0.75
10	13	50	21.93	0.1917	0.84	2.21	6.1	0.79
16	14	50	35.97	0.1955	0.88	3.85	6.7	0.78
15	15	50	35.36	0.1952	0.99	4.69	7.6	0.98
7	16	75	56.12	0.2272	0.99	6.44	7.9	0.98
6	17	75	49.20	0.2188	0.97	4.67	3.4	0.79
18	18	50	34.89	0.1956	0.89	3.81	6.6	0.73
3	19	25	31.20	0.1595	0.97	4.33	11.0	0.96

ANOVA was utilized to rigorously assess the experimental results, examining the effects of different parameters and revealing the interactions between them and the responses. The impact of individual parameters and their varying levels was assessed through a quadratic model, as detailed in Table 6, and the resulting models exhibited high statistical significance, evidenced by *p*-values for all responses being consistently below 0.0001. The significant impact of variables, especially temperature and TG polymer, on rheological behaviors is confirmed, with *p*-values below 0.0001 providing substantial evidence of their efficacy. This finding is further underscored by the minimal effect of ZnO NPs on the ‘n’, as indicated by a *p*-value of 0.2768. In contrast, the efficacy in influencing ‘n’ was observed solely with variations in TG polymer concentration and elevated temperature. Moreover, the effect of temperature was statistically significant for all measured response variables. The terms AB, AC, BC, and ABC represent the interactions among the factors. The influence of the interplay between factors A and B (AB) is pronounced across all response variables, as demonstrated by their *p*-values below the 0.05 threshold. Moreover, the interactions between factors A and C (AC) exhibit significantly low *p*-values for ‘n’ and YP, at < 0.0001 and 0.0328, respectively, highlighting their considerable impact on the rheological properties. The interaction between factors B and C (BC) impacts only the YP and does not affect the other responses. Table 6 highlights that the interaction term ABC, which involves the combined effects of elevated temperature, NPs and polymer levels, exerts the most significant influence on YP and ‘K’.

**Table 6:** ANOVA results for corresponding responses.

Source	Response	Sum of squares	df	Mean squares	F-value	*p* value
Model	YP	19.28	7	2.75	1070.85	< 0.0001significant
	PV	65.93		9.42	19.49	
	‘n’	0.0066		0.0009	28.15	
	‘K’	2729.26		389.89	82.38	
A-ZnO conc	YP	10.68	1	10.68	4151.04	< 0.0001
	PV	7.70		7.70	15.94	0.0021
	‘n’	0.00		0.00	1.31	0.2768
	‘K’	952.49		952.49	201.26	< 0.0001
B-TG conc	YP	0.0743	1	0.0743	28.87	0.0002
	PV	6.63		6.63	13.72	0.0035
	‘n’	0.0016		0.0016	47.61	< 0.0001
	‘K’	79.79		79.79	16.86	0.0017
C-Temperature	YP	1.14	1	1.14	444.18	< 0.0001
	PV	46.23		46.23	95.67	
	‘n’	0.0031		0.0031	94.05	
	‘K’	1617.73		1617.73	341.82	
AB	YP	3.21	1	3.21	1249.26	< 0.0001
	PV	4.65		4.65	9.63	0.0101
	‘n’	0.0004		0.0004	10.63	0.0076
	‘K’	31.09		31.09	6.57	0.0264
AC	YP	0.0153	1	0.0153	5.95	0.0328
	PV	0.6612		0.6612	1.37	0.2668
	‘n’	0.0014		0.0014	42.69	< 0.0001
	‘K’	3.39		3.39	0.7169	0.4152
BC	YP	0.0253	1	0.0253	9.84	0.0095
	PV	0.0613		0.0613	0.1268	0.7285
	‘n’	5.445E-06		5.445E-06	0.1637	0.6936
	‘K’	9.61		9.61	2.03	0.1818
ABC	YP	4.13	1	4.13	1606.83	< 0.0001
	PV	0.0012		0.0012	0.0026	0.9603
	‘n’	0.00		0.00	0.5964	0.4562
	‘K’	35.15		35.15	7.43	0.0197

**Fig. 5:**
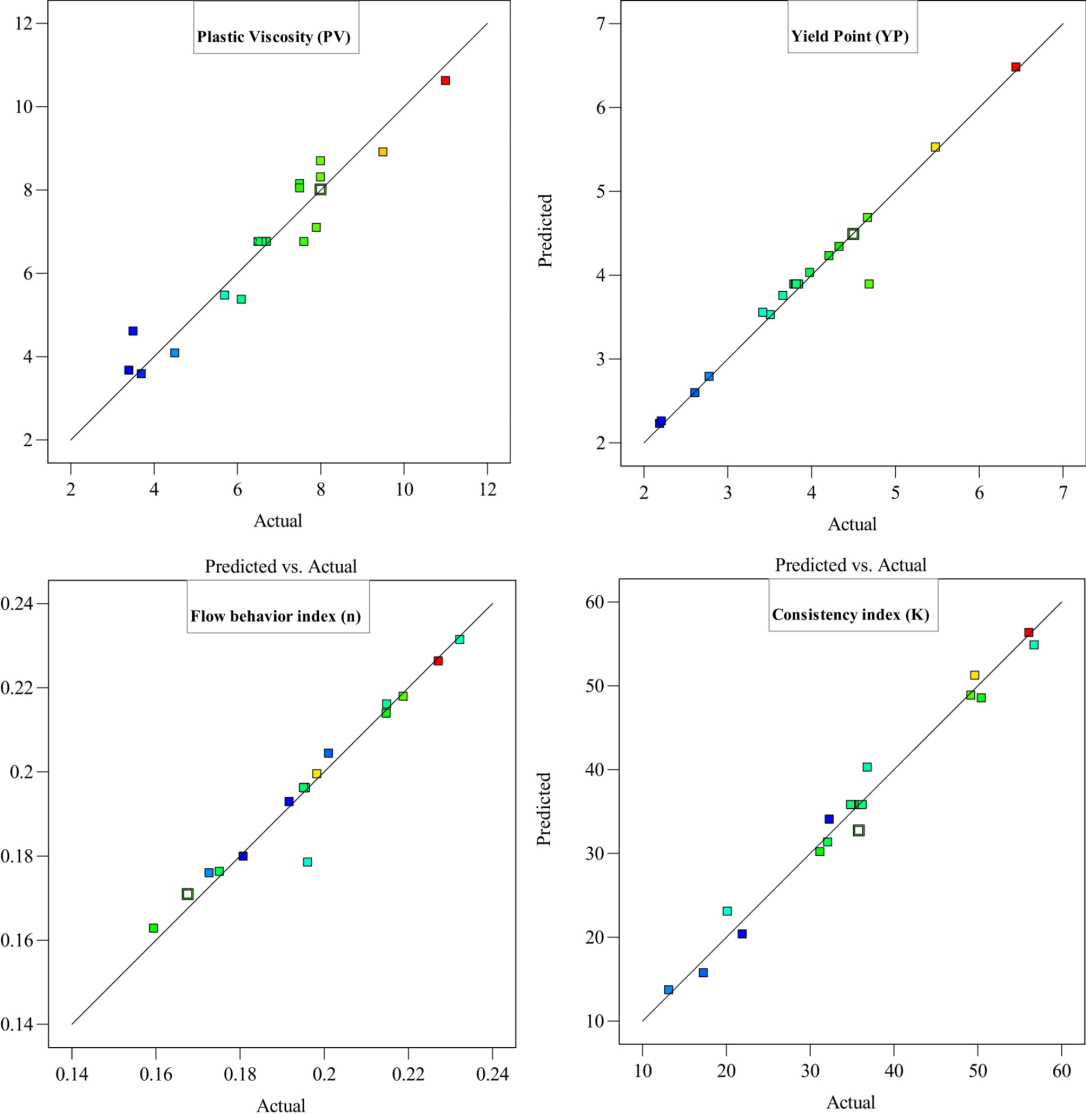
Diagnostic plots: comparison of predicted and actual values for model accuracy assessment.

The comparison between predicted and actual values for PV, YP, ‘n’, and ‘K’ demonstrate the strong predictive capability of the developed model (Fig. 5). The diagnostic plots indicate a close alignment between the predicted and actual values, with minimal deviations. This suggests that the model effectively captures the complex rheological behavior of the drilling fluid under various conditions. The PV and YP predictions exhibit high accuracy, reflecting the model’s ability to account for the influence of ZnO NPs and Tragacanth polymer on fluid resistance and shear stress. The slight variations observed are within acceptable limits, confirming the robustness of the model.

Similarly, the predicted values for the ‘n’ and ‘K’ show a strong correlation with actual experimental data. The ‘K’, which represents the fluid’s resistance to flow, is well captured by the model, reinforcing its reliability in describing non-Newtonian behavior. The ‘n’ predictions indicate accurate representation of the shear-thinning properties of the drilling fluid. The high coefficient of determination (R^2^) values further validates the model’s accuracy, demonstrating its suitability for optimizing drilling fluid formulations.

**Fig. 6:**
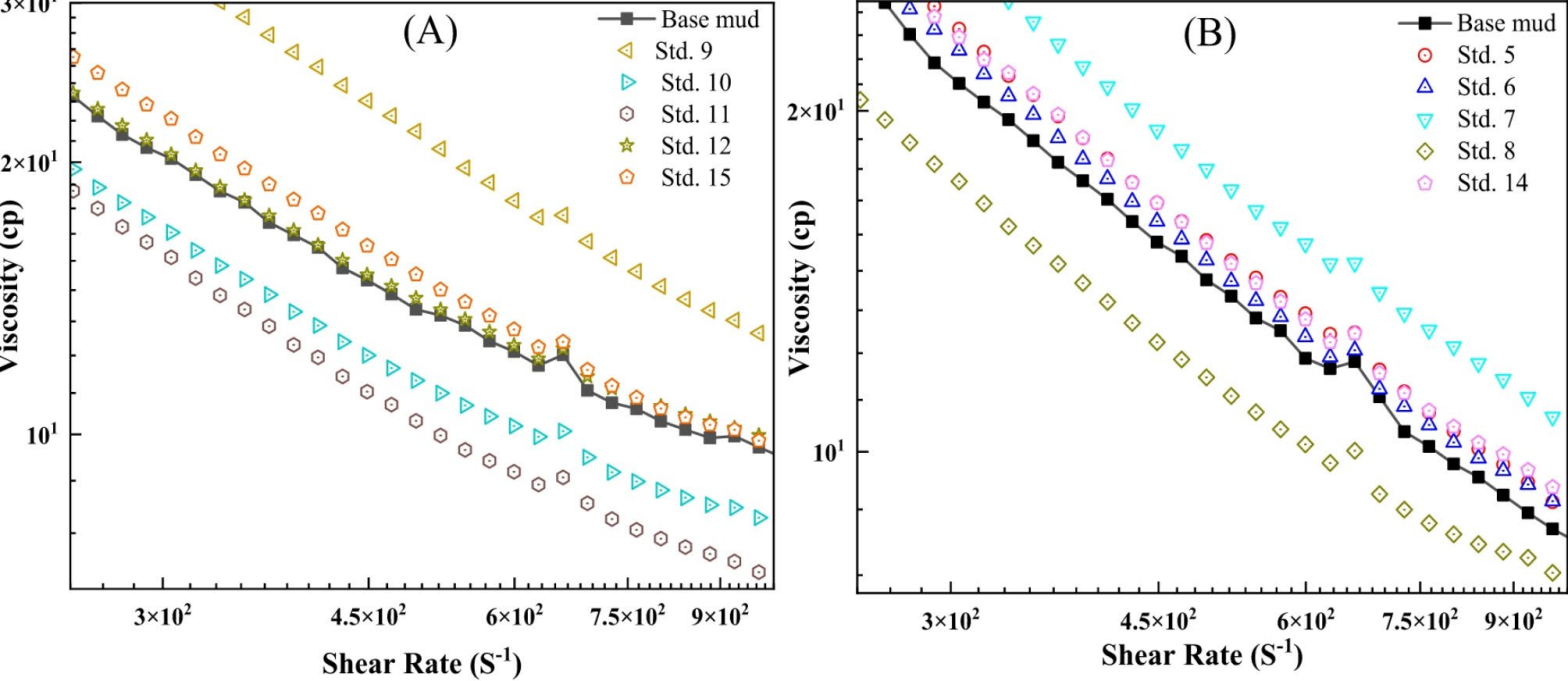
Effect of ZnO NPs/TG on viscosity of mud samples at (**A**) 50 °C, and (**B**) 75 °C.

Figure 6 illustrates the viscosity of ZnO-TG samples across various shear rates at elevated temperatures (50 and 75 °C) for different concentrations. As demonstrated, the viscosity of the mud samples diminishes as the shear rate increases, signifying a shear-thinning behavior. Moreover, viscosity was influenced by the concentration of the polymer, with viscosity rising as the polymer concentration increased. The steady shear viscosity increased as the temperature rose from 25 to 75 °C at a constant shear rate. The increase in shear viscosity with the rising temperature was ascribed to the formation of colloidal structures within the formulations. NPs increase the gel strength of drilling fluids by requiring more force to break the electrostatic bonds in fluids with higher colloidal particles. They also help maintain gel strength at higher temperatures by dissipating heat more effectively, thereby reducing thermal degradation^53^. The shear viscosity increased when a high polymer concentration was combined with a low ZnO NPs concentration in the bentonite suspension. This increase is attributed to the adsorption of polymer chains onto the negatively charged surfaces of bentonite platelets. At low shear rates, the shear viscosity increases with rising temperature, primarily due to the entanglement of polymer molecules, which enhances the viscosity. At medium shear rates, a temperature rise reduces shear viscosity due to unraveling polymer chain entanglements. At high shear rates, the increase in viscosity is attributed to the formation of bridges between ZnO NPs, TG polymer, and bentonite particles^26,67^. The combination of ZnO NPs and TG polymer enhances the rheological properties of drilling fluids, particularly under HTHP conditions. TG improves viscosity and gel strength, while ZnO NPs reinforce the polymer structure, increasing thermal stability and preventing degradation.

This synergy enhances the fluid’s viscoelastic behavior, ensuring a balanced elastic and viscous response. The polymer-NPs network improves resistance to deformation while maintaining controlled flow, leading to better cutting suspension, improved hole cleaning, and increased stability under HTHP conditions.

**Fig. 7:**
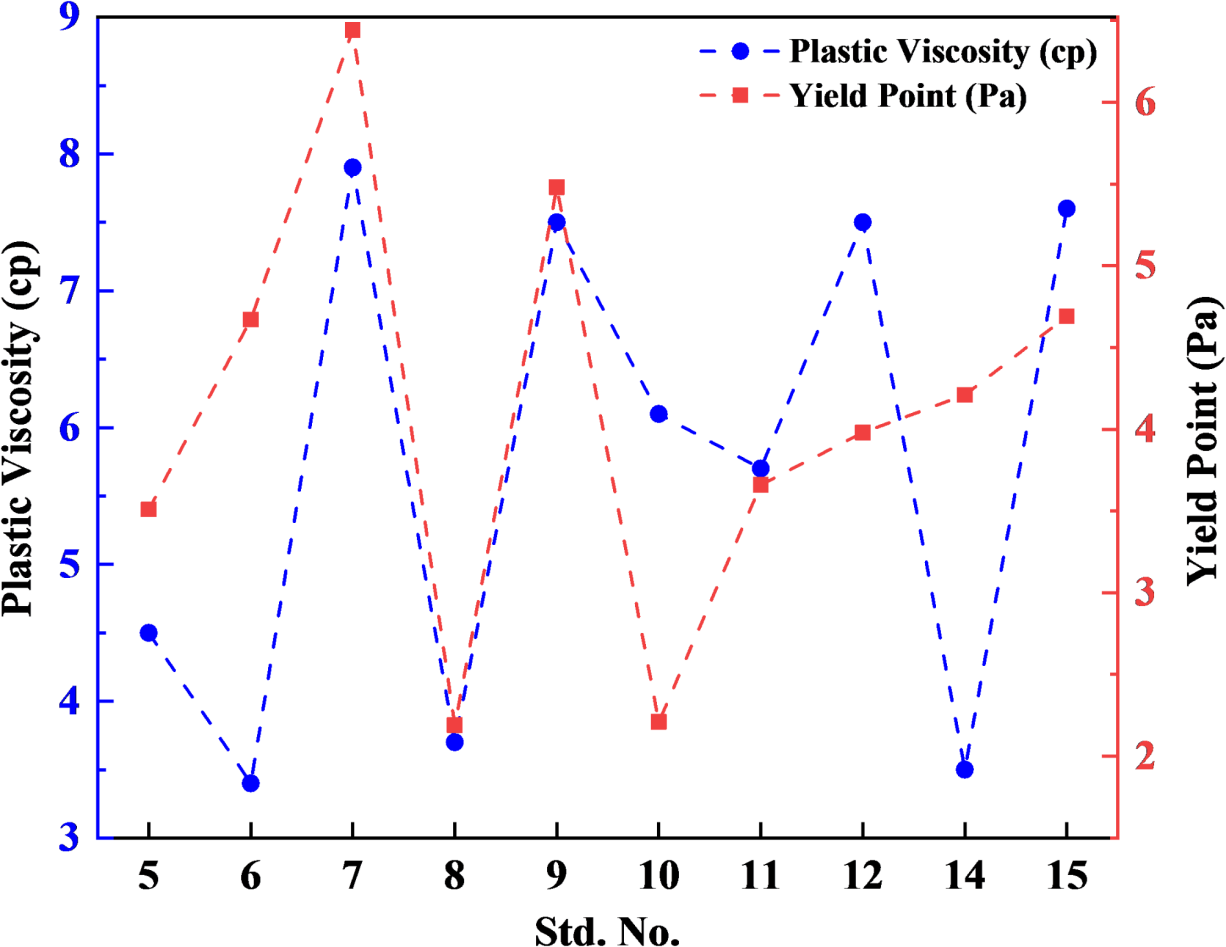
Effect of ZnO-TG solutions on YP, and PV at elevated temperatures.

Temperature significantly affects WBM stability. Higher temperatures increase particle agglomeration and decrease zeta potential, leading to WBM instability^64^. Additionally, since particles are electrically charged, a reduction in particle size can lead to an increase in PV^91^. The slight increase in the PV of the WBMs is likely due to the effective dispersion of ZnO NPs, which was achieved by the high concentration of TG polymer^70^. TG polymer served as a natural water-based surfactant to stabilize the dispersion of ZnO NPs within the base fluid^64^. Elevating the temperature first to 50 °C and then to 75 °C, starting from 25 °C, resulted in a reduction in PV, as illustrated in Fig. 7. This reduction is due to particle clustering, which increases particle size, especially in WBMs with high levels of NPs and low polymer concentrations^26^. The YP is impacted by electrochemical forces created by positive, negative, or neutrally charged particles in the mud. In a high-pH environment, ZnO NPs lose protons and gain a negative charge, enhancing their interaction with other charged particles and adjusting the mud’s YP^66^. Figure 7 depicts an increase in the YP of WBMs with temperature rise, specifically at low ZnO NPs concentrations and high TG polymer levels. This phenomenon can be attributed to the role of ZnO-TG in reducing particle agglomeration among the drilling additives, which occurs through its adhesion to the surfaces of these particles^26,70^. In contrast, when TG levels are low, an increase in ZnO NPs concentration results in higher surface loads within the suspension, leading to an elevation in the YP. Run 16 in Table 5, which includes 0.25 wt% ZnO NPs and 750 ppm TG, demonstrated significantly superior PV and YP at elevated temperature (75 °C), compared to runs two (TG = 0 ppm) and six (ZnO = 0 wt%), where the additives were evaluated independently. This highlights the unique contributions of each component. TG primarily enhances rheological properties by increasing PV and YP, while ZnO NPs stabilize and further improve viscosity through interaction with TG. The pronounced improvement observed in run 16 underscores the synergistic effect of ZnO and TG, emphasizing their combined role in optimizing the drilling fluid’s rheological performance. It was observed that Bingham YP increased by 32.76% and PV decreased by 39.24% by increasing temperature from 25 up to 75 °C^26,53,67^.

**Fig. 8:**
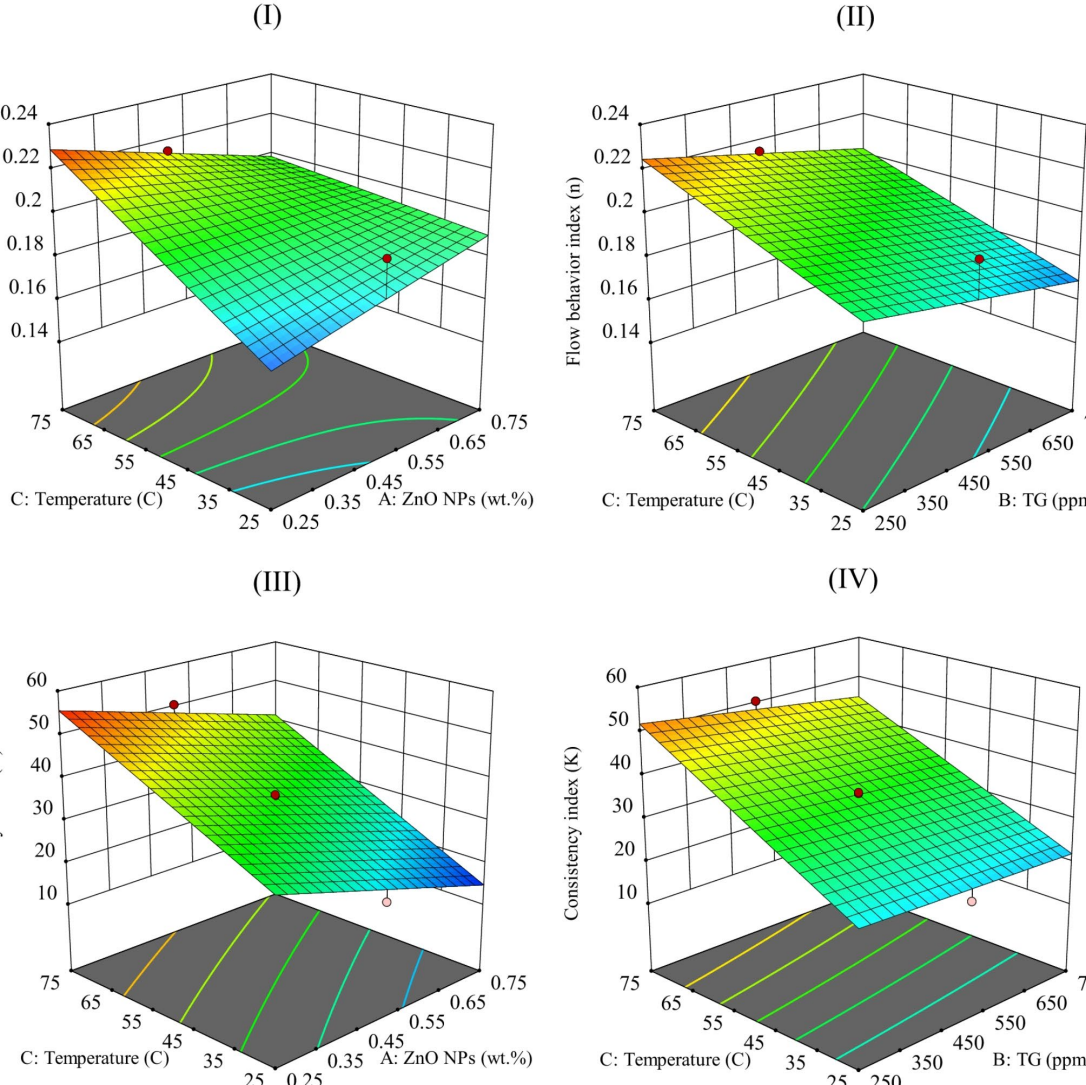
Evaluation of models using diagnostic plots, (I) and (II): effects of factors AC and BC on ‘n’, and (III) and (IV): effects of factors AC and BC on ‘K’, respectively.

As the temperature increases, the ‘n’, a power-law index indicates the shear-thinning behavior of suspensions. A lower ‘n’ indicates stronger shear thinning in the drilling fluid, improving hydraulic efficiency. The fluid exhibits lower viscosity at high shear rates and higher viscosity at low shear rates. The size of ZnO NPs and their agglomeration level are critical factors in their dispersion within a fluid. Once dispersed, ZnO NPs form groups held together by weak van der Waals forces. Thus, the adequate particle size in the fluid, known as the hydrodynamic size, is more important than the individual particle size^65^. ZnO NPs tend to aggregate at lower shear rates, but they break apart and disperse at higher shear rates. Agglomeration and deagglomeration processes contribute to the shear-thinning behavior of the dispersion^65^. Figure 8I and II show that adding ZnO NPs at low and TG polymer at high concentrations leads to more pronounced shear-thinning behavior and a lower ‘n’ value at lower temperatures. At higher temperatures and ZnO NPs concentrations, the increased size of ZnO NPs leads to reduced dispersion and a higher ‘n’ value. This issue was due to the higher size and lower surface charge of NPs in the dispersion. Higher polymer concentrations improve the ZnO NPs dispersion in the WBM and reduce the ‘n’ value. An increased ‘K’ enhances the viscosity and load-bearing capacity of drilling fluid. Improved carrying capacity contributes to more effective bottom-hole cleaning and greater drilling efficiency^65^. The interactions between ZnO NPs and TG polymer improve the polymeric network of the TG polymer and alter its structure. As a result, Fig. 8III and (IV) show a more significant increase in ‘K’ when a high concentration of TG is introduced to a low level of ZnO NPs^26,92,93^. The ‘K’ of the polymer-based WBM decreases with increasing temperature, indicating a reduced solid suspension ability at higher temperatures^94^. The ‘K’ of WBMs with ZnO NPs increases as the temperature rises, with the effect becoming more pronounced as NPs size decreases^22^. The results indicate that low concentrations of ZnO NPs and high concentrations of TG polymer enhance the shear-thinning behavior of the drilling fluid, as evidenced by a lower ‘n’. The positive effect arises primarily from the polymer entanglement, which reinforces the fluid structure. However, increasing the concentration of ZnO NPs beyond a certain point does not improve the rheological performance and may even reduce its effectiveness due to potential agglomeration or excessive interaction with other components. At elevated temperatures, the optimized formulation, which consists of low ZnO NPs and high TG polymer concentrations, improves the thermal stability of the drilling fluid by minimizing viscosity loss, as shown in Fig. 8. This combination ensures consistent rheological performance and resistance to thermal degradation under HTHP conditions, highlighting the importance of balancing the concentrations of these additives.

### HTHP filtration characteristics

**Fig. 9:**
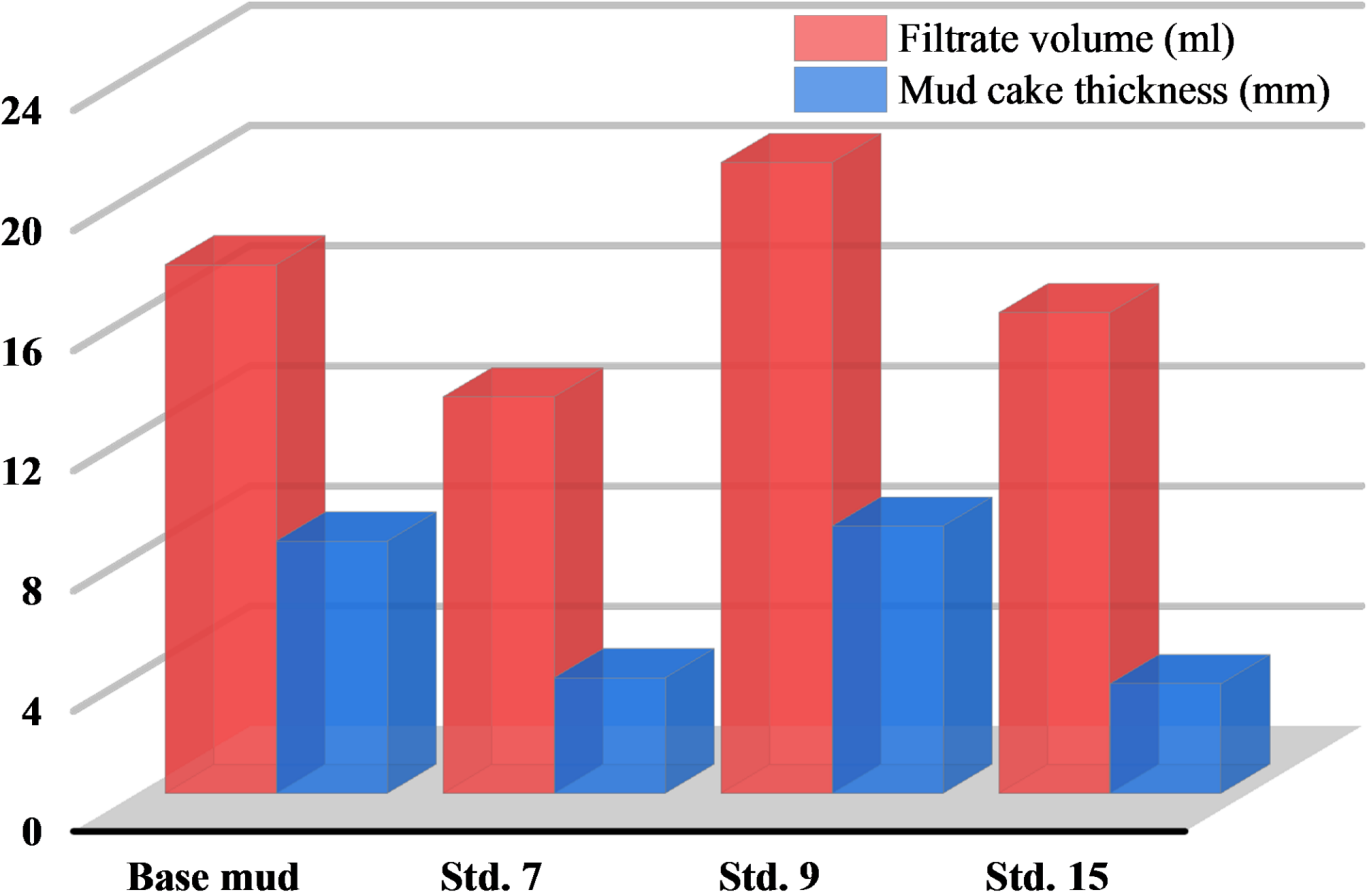
Comparison between the sample muds’ HTHP filtrate volume and final mud cake thickness after 30 min at 120 °C and 500 psi.

To develop optimal WBMs for the filtration test, RSM was employed to design 19 samples, examining the effects of elevated temperature, polymer concentration, and NPs concentration on parameters such as PV, YP, ‘n’, and ‘K’. Given the complexity of assessing the effects of NPs and polymers across varying temperatures, the ANOVA method and *p*-values were utilized to isolate the influence of each variable and identify the samples that yielded the most favorable results. Three ideal WBMs (Std. 7, 9, and 15) were found using rheological and statistical analysis findings, and each of these samples met the essential requirements needed to create an optimal WBM. The filtration characteristics of selected WBMs were examined using an HTHP filter press, at 120 °C and a pressure of 500 psi. As shown in Fig. 9, two WBMs (Std. 7, and 15) caused a significant decrease in filtrate loss and mud cake thickness compared to the base mud sample, which does not contain polymer or NPs additives. This is because the effective ZnO NPs dispersion in the base mud improved pore filling, refined the filter cake, reduced filtrate volume, and consequently decreased the mud cake thickness. The failure of Std. 9 can be attributed to the absence of ZnO NPs, unlike the other two samples. The deterioration of bentonite at elevated temperatures (~ 120 °C) is primarily caused by the breakdown of its platelet structure, the weakening of hydrogen bonds, and a reduction in its hydration capacity, leading to increased filtrate loss and weaker mud cake formation. Our findings demonstrate that the optimized concentrations of TG and ZnO NPs significantly enhance the stability of the mud system under HTHP conditions. As shown in Fig. 9, the optimized formulation resulted in reduced filtrate loss and improved mud cake integrity, highlighting the effectiveness of these additives in mitigating high-temperature-induced degradation. The ZnO NPs are functionalized and uniformly distributed throughout the filter cakes at low concentrations. They also scatter across the filter cake surfaces, filling the micropores and nanopores within the porous structure, which helps reduce the filtrate volume^3,68^. The interaction between ZnO NPs and TG polymer chains forms a network structure, with optimal performance observed at low levels of ZnO and high amounts of TG (Fig. 9). It was observed that ZnO NPs improved the thermal stability of TG polymer, positively affecting the performance of WBMs. In summary, using the formulation of (Std. 7) resulted in a filter cake that was smooth, compact, and thin. This formulation also provided excellent filtration properties and minimized fluid loss^67^. The impact of ZnO NPs on WBM rheology is primarily governed by their interactions with bentonite and TG polymer at the molecular level. The results indicate that at optimal concentrations, ZnO NPs improve viscosity and yield stress by enhancing interparticle interactions, while higher concentrations lead to agglomeration and reduced effectiveness. The correlation between rheology and filtration behavior further supports these findings, as a well-balanced formulation results in lower filtrate loss and a more compact mud cake. These results underscore the importance of carefully controlling ZnO NPs concentration to achieve optimal performance in HTHP drilling conditions.

In comparison to existing studies on NPs-enhanced WBMs, our formulation demonstrates significantly improved fluid loss control at elevated temperatures. For instance, while titanium dioxide NPs improved shale recovery and reduced filtrate loss by 27%^65^, and carbon nano tube-polymer composites reduced filtrate volume by 25%^95^, our optimized ZnO-TG formulation achieved a 54.16% reduction in fluid loss at temperatures up to 120 °C. This substantial improvement underscores the effectiveness of our environmentally friendly approach in enhancing WBM performance under HTHP conditions. These findings contribute to the advancement of NPs-polymer formulations for drilling fluid applications, offering enhanced stability and filtration control in demanding environments.

## Limitations and future perspectives of the study

Despite the valuable insights gained from this study, certain limitations should be acknowledged. The experiments were conducted under controlled laboratory conditions, which may not fully capture the complexities of real-field applications. Additionally, the study focused on a specific range of NPs and polymer concentrations, and the results may vary under different formulations or environmental conditions. Moreover, the impact of long-term stability and interactions with various reservoir rock types was not extensively explored. Future studies should consider a broader range of parameters and real-field validation to enhance the applicability of the findings.

Building upon the findings of this study, several potential directions for future research can be identified. Investigating the long-term stability and performance of NPs-polymer-based drilling fluids under dynamic reservoir conditions would provide a more comprehensive understanding of their effectiveness. Additionally, studying the interactions between these fluids and different rock formations could offer valuable insights into their adaptability in various geological settings. Furthermore, integrating advanced modeling techniques, such as molecular dynamics simulations or machine learning approaches, could help optimize fluid formulations for improved performance in drilling operations.

## Conclusion

This study highlights the potential of ZnO NPs and TG polymer as effective additives for improving drilling fluid performance under HTHP conditions. The optimized formulation enhances thermal stability, rheological properties, and environmental sustainability, making it a viable alternative to conventional drilling fluids. Key findings include:

- Synergistic effects of ZnO NPs and TG polymer: The combination enhances drilling mud performance under HTHP conditions. While ZnO NPs tend to agglomerate at high temperatures, TG polymer improves dispersion, maintaining optimal fluid properties.
- Thermal stability and rheological enhancements: ZnO NPs improve the thermal stability of TG polymer, with an optimal formulation (750 ppm TG, 0.25 wt% ZnO NPs) increasing PV by 27.84% and YP by 43.16%, while reducing mud cake thickness by 25% and filtrate volume by 54.16%. These enhancements optimize filtration and rheological performance for extreme drilling conditions.
- Environmental sustainability: The TG polymer and green-synthesized ZnO NPs minimize the use of synthetic additives, reducing environmental impact. Lower fluid loss and thinner mud cakes further enhance eco-friendliness, making this formulation a sustainable choice.
- Advantages over conventional drilling fluids: TG is a biodegradable alternative to synthetic polymers, and the green synthesis of ZnO NPs reduces toxic byproducts. This formulation offers superior performance with minimal environmental risk.
- Implications for future applications: The optimized formulation enhances drilling efficiency while supporting sustainable practices, making it ideal for environmentally sensitive drilling operations.

## Data Availability

All data generated or analyzed during this study are included in this published article.

## Abbreviations

API: American petroleum institute
ANOVA: Analysis of variance
Adj R^2^: Adjusted R-squared
BE: Binding energies
‘K’: Consistency index
CCD: Central composite design
Conc.: Concentration
DI: Deionized
EDS: Energy-dispersive X-ray spectroscopy
FE-SEM: Field-emission scanning electron microscopy
‘n’: Flow behavior index
HTHP: High-temperature, high-pressure
NPs: Nanoparticles
PV: Plastic viscosity
RSM: Response surface methodology
TGA: Thermogravimetric analysis
TG: Tragacanth gum
WBM: Water-based mud
XPS: X-ray photoelectron spectroscopy
YP: Yield point
ZnAc⋅2H_2_O: Zinc acetate dihydrate
ZnO: Zinc oxide
